# International Trade and Investment Agreements as Barriers to Food Environment Regulation for Public Health Nutrition: A Realist Review

**DOI:** 10.34172/ijhpm.2020.189

**Published:** 2020-10-19

**Authors:** Kelly Garton, Anne Marie Thow, Boyd Swinburn

**Affiliations:** ^1^School of Population Health, University of Auckland, Auckland, New Zealand.; ^2^Menzies Centre for Health Policy, The University of Sydney, Sydney, NSW, Australia.

**Keywords:** Non-communicable Diseases (NCDs), Nutrition Policy, International Trade, Investment Agreements, Policy Analysis

## Abstract

**Background:** Achieving healthy food systems will require regulation across the supply chain; however, binding international economic agreements may be constraining policy space for regulatory intervention in a way that limits uptake of ‘best-practice’ nutrition policy. A deeper understanding of the mechanisms through which this occurs, and under which conditions, can inform public health engagement with the economic policy sector.

**Methods:** We conducted a realist review of nutrition, policy and legal literature to identify mechanisms through which international trade and investment agreements (TIAs) constrain policy space for priority food environment regulations to prevent non-communicable diseases (NCDs). Recommended regulations explored include fiscal policies, product bans, nutrition labelling, advertising restrictions, nutrient composition regulations, and procurement policies. The process involved 5 steps: initial conceptual framework development; search for relevant empirical literature; study selection and appraisal; data extraction; analysis and synthesis, and framework revision.

**Results:** Twenty-six studies and 30 institutional records of formal trade/investment disputes or specific trade concerns (STCs) raised were included. We identified 13 cases in which TIA constraints on nutrition policy space could be observed. Significant constraints on nutrition policy space were documented with respect to fiscal policies, product bans, and labelling policies in 4 middle-income country jurisdictions, via 3 different TIAs. In 7 cases, trade-related concerns were raised but policies were ultimately preserved. Two of the included cases were ongoing at the time of analysis. TIAs constrained policy space through 1) TIA rules and principles (non- discrimination, necessity, international standards, transparency, intellectual property rights, expropriation, and fair and equitable treatment), and 2) interaction with policy design (objectives framed, products/services affected, nutrient thresholds chosen, formats, and time given to comment or implement). Contextual factors of importance included: actors/institutions, and political/regulatory context.

**Conclusion:** Available evidence suggests that there are potential TIA contributors to policy inertia on nutrition. Strategic policy design can avoid most substantive constraints. However, process constraints in the name of good regulatory practice (investor-state dispute settlement (ISDS), transparency, regulatory coherence, and harmonisation) pose a more serious threat of reducing government policy space to enact healthy food policies.

## Background

The past 30 years has seen a sea change of economic globalisation and liberalisation, which has opened up markets and harmonised regulations following the highly constrained world of tariffs, national protections, and restricted trade for much of the 20th century. In this policy environment the food industry pursued a path of expansion in terms of the size and power of trans-national companies (TNCs) being concentrated to major companies, especially food manufacturers producing certain types of ultra-processed foods such as snack foods and carbonated beverages, and other products high in salt, fats, and sugars. The adverse health effects of consuming such products are well-known.^1-9^ These companies and products penetrated low- and middle-income countries (LMICs) in an unprecedented way,^10-14^ coinciding with a dramatic increase in the prevalence of obesity and non-communicable diseases (NCDs) in all LMICs.^15-19^ In high-income countries, some NCDs like cardiovascular disease have reduced, but others like obesity and diabetes have increased inexorably.^15,20^ Malnutrition in all its forms is now by far the biggest contributor to lost disability-adjusted life years around the world.^21^

Such trends have prompted recommendations from the World Health Organization (WHO) for regulatory actions to prevent diet-related NCDs, including measures addressing labelling, price, marketing and nutrient composition.^22-24^ However, despite these being evidence-based, and globally agreed at the World Health Assembly, they are only sporadically implemented.^25^ This regulatory ‘failure to launch,’ or policy inertia, is often attributed to industry opposition, instilling government reluctance, and public quiescence.^21,26,27^ Part of this policy inertia may be constriction of policy space from international trade and investment agreements (TIAs).^28,29^ Policy space refers to “the freedom, scope, and mechanisms that governments have to choose, design, and implement public policies to fulfil their aims.”^30^ This concept therefore includes not only the ability or right of states to regulate, but also the range of content and restrictions that policies can cover, and the processes through which policy can be chosen, designed, and implemented. WHO recommendations are not binding, but World Trade Organization (WTO) and other free TIAs are, and have binding dispute settlement mechanisms. Commitments made under TIAs can thus constrain countries’ ability to regulate goods, services, intellectual property and investments to promote public interests (including public health and the environment) upstream from domestic policy processes.^31^

The high-profile investment dispute launched by tobacco giant Philip Morris Asia against tobacco plain packaging policy in Australia (2011) demonstrates the high stakes governments face when developing health-related product regulations.^32^ Although Australia successfully defended their regulation in international arbitration, it cost more than A$23 million (half of which was repaid by the claimant, leaving Australia A$12 million out-of-pocket),^33^ and had a chilling effect on other countries following suit.^34^ Faced with the risk of arbitration and settlement of a lost dispute, governments may abandon, alter, or fail to enforce certain policy proposals, even if made in good faith. Domestic regulatory vetting processes to mitigate such risks ie, regulatory impact assessments (RIAs), mean that ‘regulatory chill’ can occur before policy is even developed.^35,36^

 Government policies are needed to drive the transition towards food systems that are better suited to 21st century challenges, including reducing the enormous health and economic burden of NCDs. This review is designed to foster policy learning globally, to understand the constraints to policy-making created by TIAs and to what extent they can be averted through strategic policy design.

## Methods

 We undertook a review of global evidence on how TIAs have or could affect policy space for a series of food environment interventions for preventing NCDs guided by the realist review method. As such, this paper identifies the ways in which TIAs influence the policy space for key nutrition policies that aim to prevent NCDs. A deeper understanding of the mechanisms through which this occurs, and under which conditions, could inform public health engagement with the economic policy space. The findings here may also apply to strategic regulation of food industries to combat climate change and other environmental damage, and for NCD prevention through regulation of other unhealthy commodities.

Realist review is a theory-driven, interpretive approach to the synthesis of evidence on an intervention to examine what works, for whom, under what circumstances, and in what respects^37^ and has been used previously to examine complex nutrition policy questions.^38,39^ The approach is focussed on gathering and synthesising evidence of “the contextual (C) influences that are hypothesized to have triggered the relevant mechanism(s) (M) to generate the outcome(s) (O) of interest.”^40^ In the present study, we consider the intervention to be a country’s membership in/acceding to TIAs; a complex and context-sensitive intervention, because one could expect the same TIA to produce different outcomes in different country contexts and for specific policy proposals. In this study:

- **Contexts (C)** relate to the governments (local, state or regional) seeking to implement policy.
- **Mechanisms (M)** are causal forces or powers that contribute to a certain policy space outcome. This study examined any mechanism of action through which the ‘intervention’ of international trade and investment operates to influence policy space.
- **Outcomes (O)** were conceptualised as the impact on specific policy, either proposed or already in effect, in terms of whether a proposed/implemented policy was preserved, modified, delayed, compromised or abandoned.

In our analysis of the review findings, we drew on political economy analysis to examine how power and resources are distributed and contested in different contexts, and the implications for food environment policy outcomes.

The stages of realist review follow a systematic process of 5 steps: initial scoping of the literature for conceptual framework development; search for relevant empirical literature; study selection and appraisal; data extraction; analysis and synthesis, and framework revision.^41^ No major changes were made to the review process once initiated.

###  Scoping the Literature

**Figure 1:**
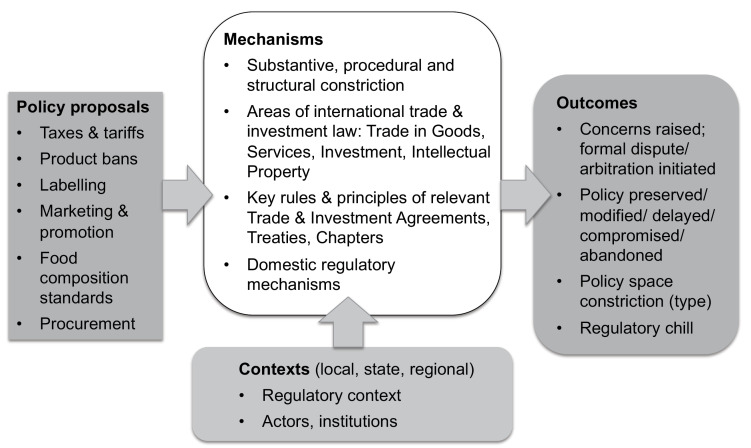
Initial Conceptual Framework for Contexts, Mechanisms and Outcomes of TIA Influences on Policy Space for Food Environment Regulations, Based on Literature Scoping. Abbreviation: TIA, trade and investment agreement.

Through a preliminary scoping of the literature related to international trade and investment, public health and NCD prevention, food environment regulation, and policy space, we identified 8 foundational sources to develop an initial conceptual framework to guide the review (see Figure 1).

**Contexts:** Voon et al^42^ suggest that political and regulatory contexts affect the impact of TIAs on policy space, including the state of evidence for need and effectiveness of proposed regulation; domestic policy and constitutional law; existing international (human rights, trade, and investment) treaty obligations; politico-economic ties, investment contracts, and import/export profiles. We hypothesized relevant agent-related factors might include: the presence and power of food-related industries; political persuasion and risk-tolerance of government; or social/cultural characteristics of the population.

**Mechanisms:** This section of the framework included any forces that influence regulatory freedoms and scope (of policy tools) substantively, procedurally and structurally^[1]^.^43^ Following Schram and colleagues’^44^ conceptual framework for investigating the impacts of TIAs on NCD risk factors, we included regulatory coherence provisions, sanitary and phytosanitary (SPS) standards and technical barriers to trade (TBT), investment chapters, and government procurement provisions as having potential influence on domestic policy space and governance for NCD prevention. We combined this with Kelsey’s^45^ outline of the legal issues underlying the various aspects of TIAs that may affect policy space for tobacco control in New Zealand: trade in goods; TBT; intellectual property rights; investor promotion, protection and enforcement; trade in services; mutual recognition; transparency and regulatory coherence. We added internal government policy process factors (eg, policy criteria, regulatory vetting procedures such as RIAs, and bureaucratic hierarchy) from Kelsey,^34^ as well as political/economic factors (eg, political will, public support, lobbying, and financial capacity) from Schram et al^46^ as potential contributors to regulatory chill.

**Outcomes:** Applying the definition of policy space from Koivusalo et al^30^ described above, we separated policy space outcomes into the types of policy space constriction described by Fidler^43^: substantive constriction, procedural constriction, and structural constriction. We note the outcomes of constriction in terms of whether a proposed/implemented policy was preserved, modified, delayed, compromised or abandoned.^46^ We considered regulatory chill^34,36,46^ a potential outcome, though with the caveat that this is difficult to observe empirically (ie, hard to observe regulatory proposals that did not proceed). We conceptualised more proximal outcomes as: whether concerns were raised to the relevant committees or governing bodies (eg, specific trade concerns [STCs] raised in the WTO TBT Committee) or formal disputes or arbitration undertaken.

###  Search Strategy

 The search for evidence included 5 academic databases covering various disciplines, 13 institutional websites, and 4 dispute databases. For feasibility, the search was limited to sources specifically covering food and/or non-alcoholic beverages regulated for public health nutrition/NCD prevention. Iterative searches were performed to settle on the best possible combination of search terms for collecting relevant results. Evidence gathered from the academic and grey literature search described above was complemented with a purposive search of evidence within trade and investment dispute databases.

###  Selection and Appraisal of Documents

Progressive screening of the academic and grey literature began with reviewing the titles of the search results for relevance to our conceptual framework (see Figure 1). Titles were screened by Author 1 (KG), and were retained if they related to:

- Nutrition policy in general, or any of the framework’s nutrition policy domains; and
- International trade and investment in general, or at least one of the framework’s mechanisms of interest; and
- Policy space (or a related term) in general, or a specific framework policy outcome.

**Figure 2:**
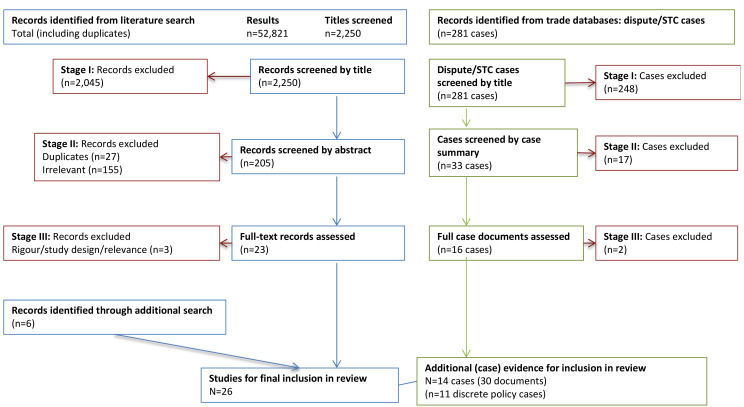
Document Search, Selection and Appraisal Flow Diagram. Abbreviation: STC, specific trade concern.

Because of the high number of results, we reviewed only the first 500-600 citations generated by each database, at which point no more relevant titles appeared (see Figure 2). The abstracts from this resulting list of retained titles were then reviewed for relevance and type of evidence, by KG with a second reviewer [BS] (See Table 1 for detail of inclusion/exclusion criteria). Once a final list of abstracts was established by both reviewers, we assessed the rigour of each full source to determine final inclusion in the review, in accordance with Realist and Meta-narrative Evidence Syntheses: Evolving Standards guidelines.^41^ We did not have to remove any sources from the final sample due to lack of rigour.

**Table 1:** Databases and Websites Searched, Search Terms and Inclusion/Exclusion Criteria

**Search**	**Databases/Institutional Websites**	**Search Terms**	**Inclusion/Exclusion Criteria**
Academic literature	Scopus, PubMed, EBSCOhost Index to Legal Periodicals and Books, Westlaw International, HeinOnline	trade OR investment - AND – food OR drink OR beverage OR diet - AND – policy OR “policy space” OR “regulatory space” OR “regulatory autonomy” OR “regulatory chill” OR concern OR dispute - AND – label* OR packag* OR warning OR tax OR ban OR marketing OR advertising OR promotion OR standard OR composition OR procurement	Studies/reports were included if: Published after 1995 in English, in a peer-reviewed journal or by an official organisation or non-government organisation with a mandate to address public health or international trade; and Identified and described factors related to international trade, policy space, and at least one of the nutrition policy domains of interest (rather than questions of domestic regulatory authority); and Involved an empirical analysis using primary sources as data; for legal papers and reports, if either interpreted case law or legal provisions, or presented theoretical research on general concepts, problems and principles related to international trade and investment law, and its impact on nutrition policy. Studies were excluded if: Non-empirical (eg, commentaries); or Focused on food safety and biosecurity, environmental or animal welfare concerns, fair trade, or other consumer preferences (eg, organic, halal, wild/farmed, free range, GMO, country of origin, hormones); or Focused only on domestic policy space or jurisdictional constraints. Rigour – credibility and trustworthiness. Studies/reports retained if: Academic literature was peer-reviewed, authors appeared to have no conflict of interest, and producers of grey literature were reputable international organisations; and Clearly articulated study methods, and stated methods were deemed appropriate to fulfil study aims.
Grey literature	Codex Alimentarius, FAO, ICSID, ICTSD, IFPRI, IISD, OHCHR, USTR, UNCTAD, WCRF, WHO, WIPO, WTO		
Dispute databases	WTO database of formal disputes	Agricultural and food (109 results); soft drinks (1 result)	Cases were included if they: Had clear public health nutrition objectives in one of the policy areas of interest (fiscal policy, product bans, regulation of advertising and marketing, labelling, food composition standards, and procurement). Cases were excluded if they: Focused on food safety and biosecurity, environmental or animal welfare concerns, fair trade, or other consumer preferences (eg, organic, halal, wild/farmed, free range, GMO, country of origin, hormones); or Had insufficient data and documentation.
	WTO TBT STC database^a^	Food (90 results); beverage (41 results)	
	UNCTAD Investment Dispute Settlement Navigator	By economic sectors: manufacture of food products (31); manufacture of beverage products (5); food and beverage service activities (1)	
	ICSID database	Food enterprise-related cases (8); 0 relevant	

Abbreviations: FAO, Food and Agriculture Organization of the United Nations; ICSID, International Centre for Settlement of Investment Disputes; ICTSD, International Centre for Trade and Sustainable Development; IFPRI, International Food Policy Research Institute; IISD, International Institute of Sustainable Development; OHCHR, Office of the United Nations High Commissioner for Human Rights; USTR, Office of the United States Trade Representative; UNCTAD, United Nations Conference on Trade and Development; WCRF, World Cancer Research Fund; WHO, World Health Organization; WIPO, World Intellectual Property Organization; WTO, World Trade Organization; TBT, technical barriers to trade; STC, specific trade concerns; GMO, genetically modified organism. * Indicates truncating to capture all variations of the word (eg, packag* captures package, packaged and packaging). ^a^ The Sanitary and Phytosanitary (SPS) STC database was excluded because all existing concerns were related to food safety, not the policies of interest for this study.

For each of the trade and investment database sources, selection for inclusion involved first reviewing case titles for relevance to the aforementioned policy areas (see Figure 2). For the titles retained, case summaries were read to include cases where the policies had clear public health nutrition objectives, and to exclude any cases related to food safety and biosecurity, environmental or animal welfare concerns, ‘fair trade,’ or other consumer preferences (See Table 1). A decision was made to exclude energy drinks because the issues in question were mainly about food safety. Finally, inclusion was determined based on the availability and clarity of full case documentation such as arbitration meeting minutes and TBT Committee meeting minutes. For each retained case, all of the available documentation (eg, Appellate Body reports, TBT Committee meeting minutes, and arbitration panel reports) were reviewed.

###  Data Extraction

Information was extracted from each study or case on: the country context, the agreement or treaty in question, the policy area(s) discussed, policy outcomes, and the mechanisms through which the policy was/was not/could be affected, paying particular attention to information that confirmed, refined, substantiated or refuted existing theories. The data analysis matrix in which we collected data was thus developed based on the provisional conceptual framework, and adapted/expanded throughout the data collection phase. We uploaded all documents to NVivo^47^ for qualitative analysis involving the coding of C-M-O factors and key themes. Finally, we reviewed sources’ reference lists for potential additional literature to include.

###  Analysis and Synthesis Processes

We used NVivo^47^ to sort and organise the data extracted from the papers in line with the C-M-O framework, and to record any key themes, factors or concepts identified through iterative reading of the literature relating to the development of theory from the provisional conceptual framework. To begin, a set of codes was assigned for each of the C-M-O factors hypothesised in the initial provisional conceptual framework. For each text source, we recorded (as relevant): contexts (eg, country, domestic policy characteristics, economic relations, import/export profile, and population nutrition profile, social-cultural factors), mechanisms (eg, specific TIAs mentioned and rules/principles invoked), outcomes (challenge status, policy status, type of policy space constriction), policy-specific factors (eg, type of policy tool, policy content factors, policy process factors), as well as any related theory or case law. While reading each source, we tagged the relevant text as it appeared, and also added new codes to this list through iterative reading of the sources. Once each of the texts had been coded, we ran queries to assess the density of each factor/theme (how often it comes up in the data), and to ascertain the relationships between codes, and coding summaries for more in-depth reading to identify embedded patterns and sub-themes.

## Results and Analysis

###  Document Characteristics

We included 26 studies or reports, and 30 institutional case documents of formal trade/investment disputes or STCs raised in this review (Figure 2). Twelve studies and 30 institutional records presented empirical evidence, from which we identified 13 cases in which TIA constraints on nutrition policy space could be observed (Table 2). Nutrition policy space constraints (O) were documented with respect to fiscal policies, product bans, nutrition labelling, and nutrient limit policies in 12 jurisdictions (C), via the following TIAs (M): the WTO General Agreement on Tariffs and Trade (GATT), the North American Free Trade Agreement (NAFTA), and the WTO Agreement on Technical Barriers to Trade (WTO TBT), and the European Community Treaty (EC Treaty). We observed significant policy space constraints (O) in 4 middle-income country contexts (C): Mexico (fiscal policy), Samoa, Thailand, and Indonesia. In 7 contexts (C: Denmark, Peru, Ecuador, Chile, Bolivia, Mexico (labelling), and South Korea), nutrition policies faced some initial resistance on the grounds of incompatibility with TIAs (M: the EC Treaty, and STCs raised under the WTO TBT), but were justified and preserved (O). STCs raised (M) against nutrition policies in Uruguay and Saudi Arabia (C) are ongoing.

**Table 2:** Identified Cases Involving Potential TIA Constraints on Nutrition Policy Space

**Contexts**	**Nutrition Policy (Year Proposed)**	**Mechanisms: TIAs**	**TIA Principles Invoked**	**Outcomes**
Ghana	Standards on fat content of meat cuts (early 1990s)	n/a	n/a	No trade concerns identified. Policy successfully implemented.^62^
Mexico	Tax on soft drinks and other beverages sweetened with sweeteners other than cane sugar (imposed 2002)	WTO GATT (1 dispute) NAFTA (3 disputes)	Discrimination (Art. III National Treatment) Indirect expropriation, fair and equitable treatment, national treatment, performance requirements	Decided in favour of complainant.^63^ Decided in favour of complainants, compensation awarded.^64-66^ Policy abandoned.^53,60,61^
Denmark	Ban on trans-fatty acids (2% limit) (2003)	EC Treaty	Restriction on the free movement of goods within the EU (EC Treaty Art. 28 and Art. 30)	EC took initial steps toward prosecution, but dropped case following Denmark’s presentation of evidence.^52,67^ Policy preserved, implemented 2004.
Thailand	Front-of-pack traffic-light nutrition label and “children should take less” warning for snack foods (2006)	WTO TBT	Rationale, legitimacy Transparency Unnecessary barrier to trade	STCs raised (6 times).^68-73^ Policy postponed (2008), significantly modified/compromised version implemented in 2013.^74-76^
Samoa	Import ban on turkey tails (implemented 2007)	Negotiated as part of acceding to WTO	Discrimination – availability of ‘like’ products Necessity – single product ban inappropriate to tackle the complex problem of obesity	Policy abandoned 2011.^77,78^
South Korea	Revision of nutrition labelling standards (2008)	WTO TBT	International standards (Codex) Unnecessary barrier to trade	STCs raised (once).^79^ Policy preserved, implemented 2009.
Mexico	Revision of nutrition labelling standards (updated Guideline Daily Amounts) (2009)	WTO TBT	Further information, clarification	STCs raised (once).^80^ Policy preserved, implemented 2011.
Chile	Front-of-pack stop sign nutrition warning label, restrictions on advertising to children (2013)	WTO TBT	Discrimination Further information, clarification International standards Rationale, legitimacy Time to adapt, ‘reasonable interval’ Transparency Unnecessary barrier to trade Other: impact for labelling of small packages, lack of scientific basis for nutrient thresholds, burdensome requirements, coverage of package surface, placing of stamp/label, cost increases, consumer misleading, availability of alternatives, short implementation deadlines	STCs raised (12 times).^81-92^ Policy preserved, implemented 2016 (with small modification to colour and size).^75,76,93-95^
Indonesia	Health warning nutrition labelling (2013)	WTO TBT	Further information, clarification International standards Time to adapt, ‘reasonable interval’ Transparency Unnecessary barrier to trade Other: adverse impact of mandatory health warnings, specifics related to testing	STCs raised (11 times).^82-92^ Policy modified, delayed until 2019.^75,76^ No evidence of an update at time of analysis (2020).
Ecuador	Traffic light nutrition label (2013)	WTO TBT	Further information, clarification International standards Rationale, legitimacy Time to adapt, ‘reasonable interval’ Transparency Unnecessary barrier to trade Other: mandatory nature of requirements, burdensome requirements	STCs raised (12 times).^84-92,96-98^ Policy preserved, implemented 2014.^75^
Peru	Front-of-pack stop sign nutrition warning label (2013)	WTO TBT	Further information, clarification International standards Rationale, legitimacy Time to adapt, ‘reasonable interval’ Transparency Unnecessary barrier to trade Other: lack of scientific evidence on nutrient thresholds, lack of cost-benefit analysis, consumer misleading, coverage of more foods and products than notified to WTO	STCs raised (14 times).^82-92,96-98^ Policy preserved after several modifications, implemented 2019.^75,76^
Bolivia	Traffic light nutrition labelling (2016)	WTO TBT	Further information, clarification International standards Rationale, legitimacy Transparency/	STCs raised (once).^90^ Policy preserved, implemented (2017).
Uruguay	Front-of-pack stop sign nutrition warning label (2018)	WTO TBT	Further information, clarification International standards Rationale, legitimacy Unnecessary barrier to trade Other: lack of scientific basis, consumer misleading, burdensome	STCs raised (3 times, ongoing).^99-101^
Saudi Arabia	Added sugar content limit in certain foods (2019)	WTO TBT	International standards Rationale, legitimacy Time to adapt, ‘reasonable interval’ Transparency Unnecessary barrier to trade Other: Insufficient scientific evidence, lack of clarity, burdensome requirements, potential negative effect on market demand	STCs raised (twice, ongoing).^100,101^

Abbreviations: GATT, General Agreement on Tariffs and Trade; TIA, trade and investment agreement; WTO, World Trade Organization; TBT, technical barriers to trade; NAFTA, North American Free Trade Agreement; STCs, Specific Trade Concerns; EC, European Community.

Fourteen studies in the remaining academic and grey literature are largely theoretical or speculative, based on in-depth prospective analysis of the text of the above and other existing agreements, including the WTO Agreement on Trade-Related Aspects of Intellectual Property Rights (TRIPS),^48^ WTO TBT,^49-52^ GATT,^49,51,53^ EC Treaty,^54^ NAFTA,^55^ Trans-Pacific Partnership Agreement (TPPA),^56–58^ United States-Korea Free Trade Agreement (KORUS),^57^ and trade agreements^59,60^ and investment agreements^48,61^ in general.

###  Outcomes: Summary of Challenges

The only formal trade and investment challenges raised to date against a public health nutrition policy have been against Mexico’s (2002) tax on soft drinks using sweeteners other than cane sugar, which was found to be discriminatory and in violation of obligations under the GATT and NAFTA.^63-66^ While the soft drink tax has the appearance of a nutrition policy, its objective was in fact retaliation for the United States’ alleged noncompliance with NAFTA obligations.^60^ The complainants’ main issue with the tax was its exclusion (ie, protection) of domestically-produced cane sugar, and thus discrimination against ‘like’ products—these being all other sweeteners, including high-fructose corn syrup from the United States.

There have also been several instances of constraints on policy space arising through other trade-related mechanisms. An import ban on turkey tails in Samoa was reversed as part of acceding to the WTO.^77,78^ One trade-related concern was the effectiveness of the ban in achieving the objective of improving diets and preventing NCDs—a complex problem—through prohibition of a single food item in the food system (questioning its ‘necessity’ in light of trade restrictiveness). Another was that other high-fat ‘like’ foods on the market were not subject to regulation (ie, potential for discrimination).^77^ Interpretive nutrition labelling policies have been subject to STCs raised in the WTO TBT Committee since Thailand first proposed one such initiative in 2006.^74,75^ Notably, this first example only targeted 5 categories of snack foods, leading WTO Members to question its rationale in light of the objective of improving nutrition; such uneven or incomplete coverage, at the same time as altering the conditions of competition, is an indication that the policy in question may not be the most effective means to address the country’s nutrition objectives. However, extensive lists of concerns have consistently been raised against the more comprehensive labelling policies that followed. None has progressed to formal disputes, but outcomes have ranged from policy being preserved (in Peru, Ecuador, Chile, Bolivia, Mexico and South Korea), to modified/compromised (in Thailand), or significantly delayed (in Indonesia) (Table 2). Discussion of STCs raised regarding Uruguay’s proposed nutrition warning labels was ongoing at the time of analysis.

Aside from an EC Treaty dispute process initiated against Denmark’s ban on trans fatty acids (TFAs) that was later dropped,^67^ there appear to be no trade- or investment-related challenges to nutrient composition regulations through mandatory TFA or sodium reduction policies.^52^ In 2019, the Kingdom of Saudi Arabia announced a plan to impose a maximum limit on added sugar for all food and beverages. This was subject to a number of STCs, including its deviation from international standards, transparency and time to adapt, being more trade restrictive than necessary, insufficient scientific evidence, and questioning its rationale and legitimacy.^100,101^ The representative of Saudi Arabia promptly clarified that this proposal would be under review until further notice, and the STC discussions were ongoing at the time of analysis.

There is evidence of trade-related arguments being raised against emerging advertising restrictions in Chile,^93,94^ though no concerns have been raised in formal dispute channels such as the WTO TRIPS Council to date. No challenges have been raised to procurement policies for public health nutrition to date.

**Figure 3:**
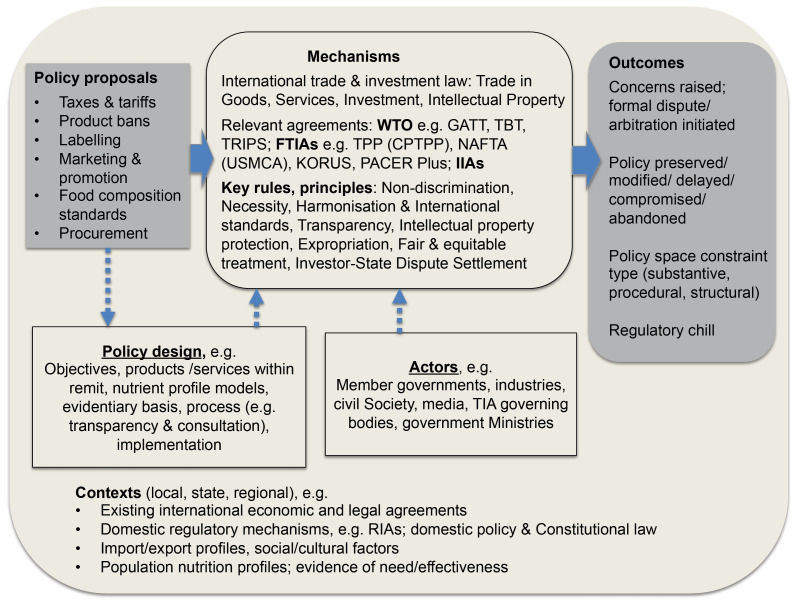
Revised Conceptual Framework for the Influence of International TIAs on Policy Space for Food Environment Regulations. Legend: Solid arrows indicate direct mechanisms, and dotted arrows show indirect mechanisms of influence. Abbreviations: TIA, trade and investment agreement; WTO, World Trade Organization; TBT, technical barriers to trade; GATT, General Agreement on Tariffs and Trade; RIAs, regulatory impact assessments; TRIPS, Trade-Related Aspects of Intellectual Property Rights; TPP, Trans-Pacific Partnership; KORUS, Korea Free Trade Agreement; NAFTA, North American Free Trade Agreement; FTIAs, free trade and investment agreements; CPTPP, Comprehensive and Progressive Agreement on Trans-Pacific Partnership; USMCA, United States Mexico Canada Agreement; IIAs, international investment agreement.

The findings from the literature reviewed are presented here, in the form of a revised conceptual framework (Figure 3). In terms of **mechanisms:** the data collected indicates that TIAs constrain policy space substantively and procedurally directly through *rules and principles* (non- discrimination, necessity, international standards, transparency, intellectual property, expropriation, fair and equitable treatment, and investor-state dispute settlement [ISDS]), and indirectly via their interaction with *policy design* factors (objectives framed, products/services affected, nutrient thresholds chosen, formats, and time given to comment or implement). This policy space is also indirectly influenced through the *interpretation and use* of TIA text, by the various actors involved, which is related to their power, resources and capacity. Relevant actors and institutions include Member governments and their various ministries, industry stakeholders, TIA governing bodies, as well as civil society and the media. Likewise, domestic and regional regulatory contexts can have a moderating effect on whether or not mechanisms of influence are activated.

###  Mechanisms

####  Direct Trade and Investment Agreement Mechanisms

Most of the literature reviewed described direct mechanisms of TIA influence on policy space in terms of the text within agreements. A majority of the reviewed literature (17/26 of the academic and grey literature, and all but one case) on policy space for priority nutrition policies to prevent NCDs discuss the implications of rules on trade (rather than investment). Trade-focused literature covered WTO and/or regional or bilateral free TIAs (which we use hereinafter to distinguish from WTO trade agreements and investment agreements), but most heavily focusing on WTO agreements (eg, GATT, TBT, TRIPS). Seven of the academic papers were concerned with more recently developed bilateral and multilateral free TIAs, that go beyond WTO obligations (which we refer to as WTO+). These included NAFTA, the TPPA, KORUS, PACER Plus, and the EC Treaty^[2]^. Investment law was less frequently discussed, with only 3 academic sources focussing on investment agreements specifically. The international investment landscape involves a complex web of thousands of bilateral investment treaties. It is also increasingly intertwined with multilateral WTO+ trade agreements, most of which will include a chapter on investment protections. Thus, the findings related to investment mechanisms presented here are by no means complete, exhaustive, nor up-to-date. It is important to note that there are also new agreements and new exceptions in some of the WTO+ agreements that were not present in the data collected.

This review indicated that the TIA rules and principles that may present direct mechanisms of constraint to policies seeking to improve food environments to prevent NCDs include non-discrimination, necessity, harmonisation/adherence to international standards, transparency/ notification, regulatory coherence, intellectual property rights, (indirect) expropriation, fair and equitable treatment, and ISDS (see Table 3). We have described these as generally as possible, and it is important to note that the rules, definitions, clarifications and exceptions associated with these general principles will differ from one agreement to another, and may be especially different in recent TIAs.

**Table 3:** Mechanisms of Influence on Nutrition Policy Space Related to Agreements

**Principle/Mechanism** **and Related Agreements**	**Articles and Exceptions Documented in Literature **	**Type(s) of Constraint and Description/Summary**	**Moderating Factors**	**Relevant ** **Policy Area(s)**
**Non-discrimination** ***MFN and National Treatment*** Found in: WTO: GATT, GATS, TBT, SPS; FTIAs; IIAs	WTO: GATT Art. 1.1 (MFN), Art. 3.2 & 3.4 (NT), General exception XX(b) and Chapeau; TBT Art. 2.1; SPS Art. 5.5	Substantive constraint. Foreign products/services/investments should receive treatment no less favourable than ‘like’ products/services/investments of domestic origin or like circumstances – both in *intent* and in *effect.* The GATT provides for exceptions to some rules where policy is necessary to protect human, animal or plant life or health^a^, with a Chapeau that specifies: as long as measures are not applied arbitrarily/unjustified discrimination and not hidden protectionism.^49–51,53,59,62^ Non-discrimination in GATS is similar to GATT and TBT obligations.^60^	Products, services and investments within remit of agreement. Products, services and investments within remit of the regulation, ie, do any similar products exist on the market, which are not subject to regulation. Determination of ‘like’ products or circumstances. Any exceptions or explicit health protections.	All
**Necessity** Found in: WTO: GATT, TBT, GATS; FTIAs	WTO: GATT Gen. exception XX(b); TBT Art. 2.2; GATS Art. 14	Substantive & Process constraints. Policy must be the least trade restrictive measure available to achieve a *legitimate* desired objective (such as health protection or to ensure quality of a service).^49,50,59,75,93^ Evidence is required to justify. For services: Additional disciplines may apply; for example, that any Technical standards should be: based on objective and transparent criteria, not more burdensome than necessary to ensure the quality of the service, and not in themselves a restriction on the supply of a service.	Availability and quality of evidence justifying need and projected effectiveness in achieving desired objective. Framing of policy objectives. Under GATS this depends upon specifically-listed service sectors (which often include Advertising through Audio-visual services).	All
**Harmonisation and International standards** Found in: WTO: TBT; FTIAs: TBT chapters	WTO: TBT Art. 2.4	Substantive and process constraints. Where an international standard exists, it should be applied as basis for regulation, except if it would be ineffective or inappropriate to do so. Codex Alimentarius is recognised as a relevant international standard for food.	Definition of what constitutes an international standard. For example, under TBT, this must include ‘open membership,’ disqualifying WHO. Codex Alimentarius guidelines. Recognition of WHO recommendations as complement/alternative to Codex.	Labelling, Nutrient composition, Advertising restrictions
**Transparency/Notification** Found in: WTO: TBT, GATS; FTIAs: Transparency chapters	WTO: TBT Art. 2.9; GATS Art. 3; TPPA/CPTPP transparency chapter; KORUS Art. 9.6	Process constraint. If a measure does not follow international standards (or no relevant standard exists), members must notify others, provide information and allow time for comment (WTO TBT). Governments must promptly publish any policy changes affecting trade in services (GATS).^59^ Deviations from WTO rules may ratchet-up these responsibilities, making them more onerous for governments and providing greater rights to industry stakeholders. Corporations may be better equipped to oppose any proposed nutrition policy. ^56,57^ The TPPA/CPTPP goes beyond WTO rules, requiring prior stakeholder consultation. This generally involves requirements to provide notice and publish information about policy and administrative changes.	Specific wording of disciplines, for instance the definition of ‘stakeholders’ or ‘interested persons.’ For example: KORUS text on transparency regarding TBT required Parties to allow stakeholders (individual or corporate) of the other party to participate in the development of standards, technical regulations and conformity assessment procedures.^57^ This introduces greater industry access into policy-making processes.	All
**Regulatory coherence** Found in: FTIAs: Regulatory coherence chapters	TPPA/CPTPP Regulatory coherence chapter	Process constraints. Aims to streamline regulation across Member countries. This novel mechanism first appeared in the TPPA/CPTPP, which prescribes consultation & coordination mechanisms that may require governments to provide opportunities for stakeholder input into policy-making. Could further prescribe how regulations are developed at the domestic level, including providing greater role and access for industry input.^56,57^	Enforcement terms. For example, under the TPPA/CPTPP Regulatory Coherence principles cannot be legally enforced, but rather are to be adhered to in good faith. However, newer FTIAs such as the USMCA do include enforcement mechanisms.	All
**Intellectual property rights** Found in: WTO: TRIPS; FTIAs: IP chapters; IIAs: IP chapters	WTO: TRIPS Art. 15; TRIPS Art. 16; TRIPS Art. 20; TPPA/CPTPP IP chapter	Substantive constraint. The nature of goods or services should not be an obstacle to registration of a trademark (TRIPS Art. 15). ‘Unhealthiness’ of a product or food service could therefore be interpreted as *not* a valid reason to restrict the *registration* of a trademark.^59^ Registered trademark owners have exclusive ‘negative rights’ to prevent its use by third parties (TRIPS Art. 16), but this does not necessarily mean they have the ‘positive right’ to use them.^59,94^ Trademarks should not be *unjustifiably *encumbered by special requirements (TRIPS Art. 20). Thus, any policy seeking to restrict use or placement of brand names, logo, licensed characters, or other distinguishing marks or design features would need proper justification.^59,93,94^ Health Impact Assessment of the TPPA (now replaced by the CPTPP) indicated that it extended the protections of trademarks (eg, on packaging) beyond those set out in TRIPS.^56^	Availability and quality of evidence justifying need and projected effectiveness in achieving desired objective. Framing of policy objectives.	Labelling, Advertising restrictions
**Expropriation (indirect)** Found in: FTIAs: Investment chapters; IIAs	NAFTA Investment chapter (Ch. 11); TPPA/CPTPP Investment chapter; KORUS Investment chapter	Substantive constraint. Expropriation of an investment, even for a ‘public purpose’ and without discrimination, may still warrant compensation. One reviewed legal analysis of NAFTA indicated that a regulatory taking (ie, expropriation) would have to be extreme in order for a claim of indirect expropriation to be upheld.^55^ However, precise wording in different investment agreements and chapters will vary, and this has yet to play out in a dispute regarding nutrition policy.	Detail of clarification. Newer TIAs may specify, eg, the ‘degree of impact on an investment’ and what constitutes non-discriminatory actions,^b^ but older BITs do not contain such clarifications. Definition of public health purpose.^c^ Similarly, newer agreements may include clearer definitions. Exceptions. General Exceptions do not apply to this chapter in the TPPA/CPTPP for example.	All (especially those involving trademarks (labelling, advertising restrictions)
**Fair and Equitable Treatment** Found in: FTIAs: Investment chapters; IIAs	NAFTA Ch. 11; TPPA/CPTPP Investment chapter	Substantive and process constraints. Its meaning is notoriously ambiguous and inconsistently interpreted, but this standard generally protects the ‘legitimate expectations’ of an investor of the regulatory environment. Thow and McGrady^61^ interpret that investors have no right to expect the regulatory environment to remain unchanged, but if a host induces investment and later introduces a policy that regulates the products of that investment, this challenge could be applied. However, precise wording in different investment agreements and chapters will vary, and this has yet to play out in a dispute regarding nutrition policy.	Interpretations of ‘legitimate expectations.’ For example, this may include previous promises made pertaining to regulatory environment, incentives given or contractual commitments made,^61^ though this is not necessarily required for an award to be made. Clarification of the definition and scope of fair and equitable treatment within agreements.	All
**ISDS** FTIAs: Investment chapters (some) IIAs	NAFTA Ch. 11; TPPA/CPTPP Investment chapter; KORUS Investment chapter	Process constraint. Allows investors to directly challenge government policy, rather than appealing to their host government to do so. Awards for compensation can include projected loss of future profits and compound interest. Investor-state arbitration processes have been criticized for being non-transparent, for lacking some of the safeguards of domestic legal processes, and for failing to consider broader issues related to public policy.^57^ The composition of dispute settlement tribunals (3 private sector lawyers) has raised concerns of bias toward industry interests.^57^	Carve-outs or exceptions from ISDS for health and/or nutrition regulations. For example, the CPTPP excludes tobacco control measures from the ISDS mechanism.	All

Abbreviations: TIA, trade and investment agreement; WTO, World Trade Organization; TBT, technical barriers to trade; GATT, General Agreement on Tariffs and Trade; TRIPS, Trade-Related Aspects of Intellectual Property Rights; TPPA, Trans-Pacific Partnership Agreement; KORUS, Korea Free Trade Agreement; NAFTA, North American Free Trade Agreement; FTIAs, free trade and investment agreements; CPTPP, Comprehensive and Progressive Agreement on Trans-Pacific Partnership; MFN, Most Favoured Nation; ISDS, Investor-State Dispute Settlement; BITs, bilateral investment treaties; IIAs, international investment agreements; GATS, General Agreement on Trade in Services; SPS, sanitary and phytosanitary; WHO, World Health Organization; USMCA, United States Mexico Canada Agreement. ^a^ Determining whether a measure is “necessary” to protect human, animal or plant life or health under GATT Art. XX(b), involves the weighing and balancing of a series of factors, including the contribution made by the measure to the policy objective, the importance of the common interests or values protected by the policy measure, and the impact of the measure on international trade.^102^ ^b^ CPTPP Investment chapter Annex on Expropriation specifies the Degree of impact on an investment: “economic impact of the action; extent to which government action ‘interferes with distinct, reasonable, investment-backed expectations’; and the *character* of government action.”^103^ (p. 9-36) Annex 9-B It further specifies that “Non-discriminatory regulatory actions by a Party that are designed and applied to protect legitimate public welfare objectives, such as public health, safety and the environment, do not constitute indirect expropriations, except in rare circumstances.”^103^ (p. 9-36-9-37) Annex 9-B Under KORUS, expropriation of a covered investment is permitted ‘for a public purpose’ including ‘measures to protect health,’ and this Agreement also has exceptions for existing ‘non-conforming measures’ (ie, policy measures that do not comply with the agreement).^57^ ^c^A footnote of the CPTPP Investment chapter Annex on Expropriation states: “regulatory actions to protect public health include, among others, such measures with respect to the regulation, pricing and supply of, and reimbursement for, pharmaceuticals (including biological products), diagnostics, vaccines, medical devices, gene therapies and technologies, health-related aids and appliances and blood and blood-related products.”^103^ (p. 9-37) Annex 9-B Notably, this definition does not include regulation of food for public health nutrition.

 Many of these key rules could apply to a broad range of policy scenarios. For example, any policy that establishes technical regulations (eg, nutrition labelling, packaging, product reformulation targets, or even some restrictions on advertising that may apply to goods) could arguably fall under the WTO TBT Agreement and TBT chapters of other TIAs, or be measures that affect the supply of a ‘service’ under trade in services agreements. Similarly, the rules surrounding the protection of investments (such as fair and equitable treatment of investors) are likely to be applicable, to some extent, to all of the policy areas of focus in this study. The same policy could therefore be affected by multiple chapters across multiple agreements that do not all have identical provisions.

 The findings of this review indicate that rules on non-discrimination, necessity, harmonisation, intellectual property, expropriation, and fair and equitable treatment present potential substantive constriction on nutrition policy space. The evidence found in this review suggest that formal appeals to these rules have mainly constrained poor policy design to date. For instance, incomplete coverage of products triggering discrimination concerns, or policy settings not well aligned with the stated public health objectives being subject to concerns around necessity (eg, the Mexican non-sugar sweetener tax, Thailand’s front-of-pack labelling scheme on 5 categories of snack foods, or Samoa’s turkey tail import ban).

 On the other hand, this review indicates that necessity, harmonisation, transparency/notification, regulatory coherence, fair and equitable treatment, and ISDS rules present a procedural (policy process) form of constriction. Necessity rules entail a burden of proof that a measure is not more trade restrictive than necessary; governments must come up with a body of evidence to back their decisions, or choose a less restrictive option. Harmonisation and standardisation rules likewise require justification of any technical regulation that deviates from an established international standard (such as the Codex Alimentarius), or in cases where such a standard does not exist. Transparency and notification rules provide the opportunity for further input into the policy-making process from industry stakeholders, thereby introducing the potential for both procedural and structural constriction of policy space. This input may be established directly, as in the case of the TPPA (now replaced with the Comprehensive and Progressive Agreement on Trans-Pacific Partnership, CPTPP) and KORUS, or indirectly through their respective government representatives (eg, at TBT Committee meetings). Regulatory coherence introduces new norms into the policy-making process, which may include stakeholder consultation and involvement as part of consultation and coordination mechanisms—again, introducing the potential for procedural and structural policy space constriction (though not legally enforceable under the TPPA/CTTP). Investors may take advantage of ambiguous fair and equitable treatment standards to pursue claims against food environment regulations, and may do so even more easily through ISDS mechanisms.

####  Indirect Mechanisms

 The studies and cases in this review indicate that binding TIA rules can be understood as directly constraining policy space for food environment regulations, but that these constraints occur via their interaction with policy design factors. This policy space is also indirectly influenced through the interpretation and use of TIA text, by the various actors involved.

###  Policy Design

 This review found that policy design elements with the potential to influence policy space through interaction with trade rules include: the framing of objectives, choice of products/services affected or nutrient profiles used, and the scientific basis and evidence used to justify technical regulations. As noted previously, shortcomings in policy design were associated with nutrition policies in Mexico, Thailand and Samoa that were constrained via TIA mechanisms. Trade database records for at least 3 cases (Ecuador, Chile and Saudi Arabia) showed that factors related to ease and cost of implementation may also trigger STCs in terms of regulations being overly ‘burdensome.’ The sources reviewed indicate that, generally, strategic policy design in these areas can limit substantive TIA constraints.

Two studies^57,75^ suggested that policy design process factors of following ‘good regulatory practice’ are related to procedural constriction of nutrition policy space. Failure to notify (and in some cases, engage) trade partners and investors of regulatory proposals, and with sufficient lead time, may be flagged as a violation of TIA commitments in terms of transparency and early notification, as was the claim in 7 of the 10 STC cases we reviewed. Such notification and engagement, however, introduces greater potential (and time) for a wide range of stakeholders to influence policy, effectively using TIAs to serve their own interests.

‘Good regulatory practice’ also implies evidence-based policy design, with justification for necessity, and projected effectiveness in achieving desired policy objectives. Analysis of the literature on TIAs and nutrition policy space raises the question: *how much justifying evidence is enough?* In the context of Indonesia, proposed mandatory labelling (including warning labels) for sugar, fat and sodium content on processed and fast foods to better inform consumers about nutrition and prevent NCDs was met with STCs from several members between 2013 and 2016, questioning the scientific justification and urging consideration of alternative approaches.^76,82-92^ It was announced that the policy would be delayed 4 years while the government considered alternate approaches, but no update was evident at the time of analysis. The labelling requirements, however, were based on the Balance Nutrition Guidelines and related 2008 WHO recommendations, as well as data from a 2014 nutrition survey conducted by the Ministry of Health.^75^

###  Application and Interpretations of TIA Text

 Two studies highlighted the ways in which TIAs as structural instruments are used and interpreted to raise concerns or launch disputes, or to respond to them, constituting another indirect mechanism of constraint to policy space. Such indirect constraints relate to the power dynamics between actors involved, and their capacity to influence outcomes.

Barlow et al^76^ found that power asymmetries exist between the countries raising STCs in the TBT Committee and those responding to them, indicating that countries may use this forum as a means to exert and translate such power asymmetries into policy leverage. Wealthier nations (and companies in the case of ISDS) may have greater legal capacity to find ‘loopholes’ within agreements and use them to their advantage. The authors observed that more than 3 quarters (77.4%) of STC challenges raised against low- and lower-middle–income countries for NCD prevention regulations had been raised by wealthier nations.^76^ Another study reported that power imbalance between small Pacific Island Countries (PICs) and larger trading partners was a perceived factor constraining nutrition policy space: both in terms of influencing small PICs to enter into agreements to begin with (eg, through aid-dependency), and through the formal avenues of influence thereafter (eg, WTO rules limiting PICs’ ability to restrict imports for public health reasons).^78^

 Background literature suggested that indirect mechanisms of constraint on policy space for food environment regulation may play out even earlier in the policy cycle, for example through the domestic RIA process mentioned previously, though such a phenomenon was not reported in the data reviewed. The interpretations of trade ministries of how nutrition policy proposals would interact with trade commitments, including the lobbying of industry stakeholders to this effect, may contribute to whether or not policy moves forward to the notification stage, in which instance it would not appear as a potential case.

###  Contextual Factors

 This review indicated that contextual factors potentially influencing TIA-related nutrition policy space include the actors and institutions operating in trade and nutrition policy spheres, as well as national political and regulatory contexts.

###  Actors and Institutions

Actors may use several mechanisms to influence outcomes in their favour either directly or indirectly (eg, through the use of institutions), visibly or invisibly.^104^ This review found evidence that actors and institutional structures at both the international and national level can influence policy agendas, the power to engage in disputes, and the interpretation of evidence. Particularly relevant are government ministries, civil society organisations, food industry structure and power, consumer groups, dispute settlement bodies, and standards setting bodies.

Government departments (eg, ministries of health, trade, economy) are the principal environment in which policy is proposed and developed, and where regulatory chill occurs or does not. Five reviewed studies reported that the involvement of a broad collection of government actors (within both trade and health sectors) in agenda setting and policy development can be a supportive factor for nutrition policy space.^62,75,95,105,106^ For instance, early engagement with trade policy-makers can help to identify any easily resolvable trade concerns before the notification stage.^75^ The outcome of such engagement hinges, however, on the overall support for regulation from within the trade departments, and the type of government in power and their ideological leaning (eg, in favour of more or less government intervention in markets).^55,57^ The engagement of civil society organisations can also contribute to the constriction or opening of policy space for public health nutrition through applied pressure for regulation and holding governments accountable.^57,95^ Public support for regulation was found to have supported positive policy space outcomes for nutrition regulations in Ghana and Denmark,^62,67^ to which media attention can make a strong contribution.^67^ Consumer-citizen activism may counter-balance corporate influence into the regulatory process and help to legitimise non-discriminatory policies made in good faith for public purposes such as NCD prevention^55^ – but only when these debates happen publicly. Trade partners may also influence governments’ nutrition policy space invisibly through bilateral political relationships such as international aid,^78^ as might investors through contribution to gross domestic product.^57,61^

Food and beverage companies form a powerful interest group with a number of avenues of influence (direct, indirect, and invisible). The review indicated that the types of food industry present within a regulating country, their level of vertical investment^61^ and contribution to the economy, and the existing capacity of industry to engage in political processes can shape the policy space for public health nutrition.^57^ Commercial stakeholders may try to leverage their economic power directly by lobbying governments. Through submissions to governments during the TPPA negotiation process, for example, we know that food and beverage industry groups actively sought to shape the content of the Agreement (pushing for increased market access for processed foods, greater regulatory harmonisation, enhanced investment protection and legal remedies), particularly regarding SPS and TBT sections.^58^ TNCs can also exert invisible influence on nutrition policy space, for example through major contribution to employment and thus being a priority for government.^51,55,57^ We can infer that the stronger the industry—in terms of significance to the domestic (including export) economy and its size and lobbying power—the more likely governments may be to fight for that industry’s position in agreement negotiation. Finally, this review suggests that TNCs are able to influence nutrition policy space indirectly through strategic engagement with the institutions involved in TIA governance.

The institutions involved in setting the ‘rules of the game,’ including dispute-settlement and standards-setting bodies, have the formal authority written into Agreements to influence policy space, and the extent of this influence is both visible and invisible. WTO bodies’ proceedings are transparent (with the Appellate Body providing a means of appeal to decision-making), but many WTO+ dispute settlement bodies are not. Given their position of influence, the system of investment dispute arbitration panels has been criticised for lack of transparency and potential bias towards private sector interests, in particular due to the composition of these panels (3 private sector lawyers).^57^ The Codex Alimentarius Commission (Codex) jointly established by the United Nations Food and Agriculture Organization and the WHO is widely recognised as the body deemed appropriate for producing ‘relevant international standards’ for food and beverages, especially within the realm of food safety. For instance, Codex standards were referenced by Member representatives in each of the cases we reviewed regarding nutrition labelling. It is widely known, however, that the Codex Commission’s membership structure allows Member country delegates to invite industry representatives, and includes ‘non-government organisation’ Observers of which industry groups make up a large proportion.^95,107^ Commercial influence in this forum, eg, influencing the standards at Codex that are then used to interpret and determine necessity under TIAs, is essentially ‘mobilising the bias’ present in these institutions toward economic interests, to influence policy space in their favour.^104^

###  Political and Regulatory Contexts

This review identified several national political and regulatory factors that may influence nutrition policy space in different ways (Table 4). We found that national regulatory factors with potential to influence interpretations of policy proposals with respect to TIA rules included: availability and quality of evidence (associated with research capacity and budget), which may influence interpretations of necessity and justification^50,62,67,75^; regulatory frameworks (eg, being part of a comprehensive suite of interventions), which may also influence interpretations of justification^50,54,59,75,77,95^; and history of regulation, which may influence interpretations of ‘good faith,’ necessity, and fair and equitable treatment.^61,62,67^

**Table 4:** Political and Regulatory Context Factors Identified in Review

**National Regulatory Factors that Potentially Influence Interpretations of Policy Proposals with Respect to TIA Rules**	
Availability and quality of evidence	Influences interpretations of necessity and justification. This includes data on their stage in the nutrition transition, burden (or double/triple burden) of malnutrition, and clear need to address diet-related NCDs^62^ as well as strong evidence of the health risks associated with consumption of products to be regulated,^50,67^ and projected effectiveness of the proposed measure in achieving the objective of improving diets and preventing NCDs.^75^ WTO TBT Members questioned the scientific evidence backing the policy in half (5/10) of the STC cases reviewed.
Regulatory framework	May influence interpretations of justification. Six studies reported that a policy measure may be more robustly defensible if it is part of a comprehensive suite of interventions, including less trade restrictive alternatives such as public education campaigns (mitigating the argument that less trade restrictive alternatives are available).^50,54,59,75,77,95^
History of regulation	Can influence interpretations of good faith, necessity, and fair and equitable treatment. Long-standing history of food regulation in Ghana may have supported policy space for Ghana’s import standards on fatty meats, by setting a precedent for further nutrition regulations.^62^ Denmark’s TFA ban may have had more policy space because the measure started out as a voluntary agreement, in terms of having evidence to show that voluntary measures had been insufficient to achieve desired public health objectives.^67^ Domestic conditions surrounding incentives and contractual commitments previously given to the private sector may affect nutrition policy space with respect to investment agreements, as these may serve to establish investors’ ‘legitimate expectations’ of the regulatory environment.^61^
**National Stakeholder Factors that Potentially Influence Power Dynamics and Capacity to Influence Policy Space**	
Party to which agreements, and with whom	Power dynamics with trade partners influence governments’ relative negotiating power, and relative capacity to mount or respond to a dispute. For instance, Fa’alili-Fidow et al^78^ suggested that PICs had a weaker trade bargaining position with respect to larger, wealthier neighbours on whom they rely for aid. Barlow et al^76^ noted that more than 3 quarters of the STCs raised against LMIC public health policies in the TBT Committee were by high-income countries.
Economic stakeholder landscape	The size and importance of different private sector stakeholders (including foreign direct investment) relate to the power of an industry within country to influence government to act (or to act on its own in the case of ISDS).^55^ Vertical investment in the food supply chain gives a company greater power within a country’s food system, and increases the cumulative effect that a policy intervention may have on a given investor’s interests and their motivation and capacity to contest it.^61^
Activity and influence of civil society	The capacity and resources of civil society to engage in the policy process may influence regulatory chill. Having strong support from CSOs to advocate for health policy, generate supporting evidence, hold governments accountable, push for transparency in the policy process, and generally counter-balance industry influence, has the potential to reduce regulatory chill.^95,105,106^ Conversely, industry opposition tactics may include donating to CSOs to encourage them advocating against nutrition regulations such as marketing restrictions.^106^
**National Institutional Factors that Potentially Influence Policy Space and Regulatory Chill**	
Capacity for inter-sectoral collaboration within government	Three studies suggested that institutional structures enabling collaboration between trade and health sectors (eg, ministries or departments) in policy design could increase the capacity of governments to assess the legal basis or implications of any threats made, and reduce regulatory chill.^62,75,95^ Conversely, internal vetting processes for nutrition policy proposals in which trade and economic departments dominate may increase systemic regulatory chill.^36,57^
Financial and legal capacity within TNCs	TNCs’ institutional capacity to engage in domestic health policy-making processes (eg, through lobbying) may contribute to regulatory chill.^95^ TNCs’ capacity to engage in trade and investment dialogue and processes and to mount challenges may constrict nutrition policy space, and may increase regulatory chill in a normative sense if such challenges are successfully raised.

Abbreviations: LMIC, Low- and middle-income country; NCDs, non-communicable diseases; WTO, World Trade Organization; TBT, technical barriers to trade; STC, Specific Trade Concern; TFA, trans fatty acid; ISDS, Investor-State Dispute Settlement; TNCs, trans-national companies; CSOs, civil society organisations; PICs, Pacific Island Countries.

The literature reviewed suggested 3 national stakeholder factors that potentially influence power dynamics and capacity to influence policy space. Firstly, the set of Agreements a country is Party to, and with whom, including its power dynamics with these trade partners, may influence governments’ relative negotiating power, and relative capacity to mount or respond to a dispute.^76,78^ Second, a country’s economic landscape—and the associated size and importance of its various private sector stakeholders—relate to the power of industry stakeholders within country to influence government to act (or to act on their own in the case of ISDS).^55,61^ Third, the capacity and resources of civil society to advocate for health policy, generate supportive evidence and hold governments accountable may counter-balance industry opposition in policy-making processes, helping to reduce regulatory chill of nutrition policy proposals.^57,95,105,106^

Finally, this review indicated two national institutional factors that potentially influence policy space and regulatory chill. Three studies suggested that institutional structures within government enabling collaboration between trade and health sectors in policy design could increase the capacity of governments to assess the legal basis or implications of any threats made, and reduce the potential for regulatory chill.^62,75,95^ Within the private sector, on the other hand, individual TNCs represent institutions with potentially strong financial and legal capacity to influence policy space. TNCs’ institutional capacity to engage in domestic health policy making processes may contribute to regulatory chill,^95^ while their capacity to engage in trade and investment dialogue and processes, and to mount challenges, may constrict policy-space, and increase regulatory chill in a normative sense if challenges are successfully raised.

## Discussion

This realist review has provided greater nuance to our understanding of the ways in which TIAs may constrain policy space for priority food environment regulations. The impact on policy **outcomes** has ranged from policy being essentially preserved (eg, TFA ban in Denmark, and nutrition labelling in Peru, Ecuador, Chile, Bolivia, Mexico and South Korea), to modified or compromised (eg, nutrition warning labels in Thailand), significantly delayed (eg, nutrition warning labels in Indonesia), or abandoned altogether (eg, the non-cane sugar sweetener tax in Mexico, and turkey tail ban in Samoa). In addition to specific threats or concerns raised in trade and investment forums, policy-makers’ knowledge and understanding of such constraints likely also contributes to domestic regulatory chill, though no empirical evidence was found to this effect. Most substantive constraints on policy space (ie, when trade or investment agreements directly limit the range of policy instruments available to governments) appear to be avoidable through strategic policy design. In this sense, our findings align with the perspective Crosbie, Carriedo and Schmidt published earlier this year urging governments not to be deterred by threats from TNC trade associations to pursue challenges to evidence-informed nutrition policies through the WTO and regional trade agreements.^108^ However, procedural constraints (ie, when the process of policy-making is limited or influenced) are often linked to the incursion of influence from private sector interests in policy-making, including self-serving interpretations and use of TIA rules, and appear to be far more insidious. Increasingly, ‘good regulatory practice’ entails conducting domestic RIAs of policy proposals by relevant ministries, including departments of trade. The new generation of TIAs increasingly codify the adherence to this type of approach to policy-making. This introduces greater potential for economic stakeholders to use and interpret TIAs to serve their own interests.

 The potential influence of TIAs on policy space is different for each policy area. For instance, the evidence collected suggests that some fiscal policies (for a public health objective, if well designed and not discriminatory) appear to be compatible with trade and investment rules. Product import bans, on the other hand, are not viable under TIAs, especially if similar products exist domestically. No challenges have been raised against food procurement nutrition policies thus far. Challenges to mandatory nutrient limits/reformulation policy have been limited to date, but our findings indicate that limits on sodium or TFAs may be more viable than sugar content limits. Labelling (especially front-of-pack interpretive nutrition labelling) and restriction of advertising and marketing appear to have the greatest risk of trade- or investment-related challenges, especially in relation to emerging areas of regulating digital sales and marketing.

 Overall, the available evidence indicates that robust policy design should create sufficient policy space for regulation to achieve healthy food environments without substantive constraints from TIAs. Procedural and indirect constraints are less visible and less certain, though a willing government cognisant of the principles underlying TIAs (such as non-discrimination, necessity, etc) and thus their intersection with food system policies should be able to defend nutrition policies serving legitimate objectives.

However, this study also highlighted the power dynamics (including political capital, economic resources, and legal capacity to understand and interpret agreements) that shape how TIA texts are designed, used and interpreted. Four main dynamics stand out as contributing to policy space constraints in relation to food systems and nutrition. First, there is an underlying power asymmetry between Members in the negotiation and writing of TIAs. This has been observed, for example, from the perspective of PIC policy-makers in trade bargaining with their larger Pacific neighbours on whom they rely for aid.^78^ Second, power asymmetry exists between Members and between governments and commercial stakeholders in the interpretation and use of TIAs to further economic interests. This is both in terms of their respective resources and legal capacity to raise concerns,^76^ and their avenues of recourse to use TIAs as structural instruments.^55,57,60^ For instance, only foreign investors can challenge governments in ISDS, not vice versa,^31^ and TNCs have the option of forum-shopping to find jurisdictions where their subsidiaries have most favourable trade and investment rights. Third, there is incursion of private sector interests in institutions governing trade and investment disputes, as well as standard setting.^57^ This dynamic is particularly visible in the context of standard setting at the Codex Alimentarius Commission.^95,107^ Fourth, the underlying asymmetry between the influence of Trade and other economic Ministries in relation to Health Ministries in the policy-making process contributes to systemic regulatory chill.^36^

In line with global commentary on the agency that policy-makers and negotiators have to shape agreements and develop mitigating policies to protect domestic policy space,^31^ this research indicates 4 opportunities to preserve policy space for public health nutrition objectives. First, while this review identified ISDS provisions as potential constraints to nutrition policy space, there is global concern regarding this inclusion that has led to formal global discussions of reform as well as reduced adoption of these clauses.^109,110^ For example, the carveout for tobacco from the ISDS provisions in the CPTPP was carried over in a revised TIA between Australia and Singapore. This was subsequently significantly expanded in the Australia-Peru TIA to exclude health policy more generally from ISDS. These changes were brought about by pressure from health advocates and from governments reacting to claims over health policies, showing that this is in fact a dynamic negotiation where nutrition policy space could be regained in response to strategic nutrition advocacy. The current discontent with the international trade and investment architecture indicates a potential window for broad reform.^111^

Second, the procedural (policy process) constraints identified here suggest an opportunity to examine more closely the stages within the policy-making process at which it is appropriate that commercial stakeholders are consulted, such as in matters related to implementation.^112^ This also means clarifying consultation versus being at the policy-development table. Consultation guidelines should include management of commercial conflict of interest (which would involve careful scrutiny of industry-generated evidence and biased arguments). In addition, fair and equitable treatment clauses may more narrowly establish what legitimate expectations of investors are. For instance, in terms of transparency and consultation, it should be made clear that the scope of fair and equitable treatment does not include involvement in decision-making in line with government objectives to avoid conflict of interest. While the Framework Convention on Tobacco Control includes clear provisions regarding exclusion of the tobacco industry from tobacco control policy, demonstrating the feasibility of limits, such a hard-line approach is unlikely for regulating food and non-alcoholic beverages, as the nature of potential harm these products present is not so straightforward.

Third, this review has identified the inclusion of commitments to regulatory coherence as a cause for concern; although in many ways positive, they can be readily weaponised by powerful agents to force less powerful countries to cede policy space. This is particularly acute with broad commitments to regulatory coherence, for example, NAFTA was renegotiated to the United States Mexico Canada Agreement (USMCA) in 2019, with more far-reaching implications for regulatory coherence.^113^ This included heavy and enforceable regulatory coherence regulations, codifying the RIA-style, light-handed approach (presumption of self-regulating markets), as well as industry participation. Regulatory coherence and RIAs are being discussed in WTO plurilateral negotiations, relevant to both investment facilitation and domestic regulation disciplines. Governments wanting to preserve policy space for public health nutrition should avoid the USMCA approach of detailed, extensive and prescriptive commitments to regulatory coherence, and its use of binding language throughout.^113^

Fourth, the review indicated that international standards can either preserve or constrain policy space for nutrition depending on their quality, comprehensiveness and (freedom from) commercial conflict of interest. This suggests an opportunity to strengthen nutrition standards as international reference points, as well as their use for harmonising trade in goods. For example, if the current discussion on front of pack nutrition labelling at the Codex Commission results in a recommendation that supports strong and contextually relevant public health labelling, it could provide a strong justification for national labelling measures that are currently subject to STCs at the WTO.^107,114^ Finally, governments could routinely conduct Health Impact Assessments or Human Rights Impact Assessments for TIAs prior to or during the negotiation phase,^56^ which explicitly consider food environment policy space as part of a broader conception of the right to food security and nutrition.

###  Strengths and Limitations

 The sources reviewed span 5 academic databases covering different disciplines, two institutional TIA databases, and grey literature from all of the major international organisations working in this area. The inclusion of specific search terms for each of the policy areas of focus allowed for more detailed evidence to be gathered. The main limitation of this study is its confinement to published material, which is slow to catch up with developments in the trade and investment space. In addition, this review’s exclusive coverage of English language publications is another possible limitation. Policy innovation in these areas also still in relatively early stages, so there is a lack of empirical evidence of trade/investment barriers. Theoretical constraints (eg, in trade in services, which is largely missing in the literature but central to advertising and marketing restrictions) that might arise in the future have therefore been mostly left out.

 This study also relies on transparency. Only the WTO makes publicly available its committee discussions and arbitration decisions. Much bilateral and regional negotiation happens behind closed doors. Furthermore, new agreements are constantly being negotiated (mostly in secret), so it is unclear what their potential implications may be for public health nutrition. We note that these agreements form a dynamic space. Recent agreements tend to impose greater constraints in areas such as TBT and SPS, as well as services, although many also include clarifications to safeguard public health.

## Conclusions

 This study examined the extent to which TIAs can and have constrained governments seeking to regulate their food environments. Available evidence suggests that there are potential TIA contributors to policy inertia on nutrition, but that strategic policy design can avoid most substantive constraints. However, process constraints in the name of good regulatory practice (in the form of ISDS provisions, transparency, regulatory coherence, fair and equitable treatment, and harmonisation) pose a more serious threat of reducing government policy space to enact healthy food policies. We found that the capacity and resources of relevant actors has a moderating effect on whether such policy space constriction occurs or not (ie, whether TIA mechanisms of constraint are activated), and that there are opportunities for strategic action to mitigate potential impacts. This conceptual framework on how TIAs may constrain policy space for nutrition regulation can also provide insights relevant to measures for food system sustainability and other areas of public health.

## Acknowledgements

 The authors wish to acknowledge the comments given by Jane Kelsey on draft versions of this paper.

## Ethical issues

 Not applicable.

## Competing interests

 Authors declare that they have no competing interests.

## Authors’ contributions

 All 3 authors contributed to study conception and design, analysis and interpretation of data, and critical revision of the manuscript. Acquisition of data and drafting of manuscript was done by KG. AMT and BS provided supervision.

## Authors’ affiliations

^1^School of Population Health, University of Auckland, Auckland, New Zealand. ^2^Menzies Centre for Health Policy, The University of Sydney, Sydney, NSW, Australia.

## Endnotes


^[1]^ Substantive constriction: Occurs when trade or investment agreements directly limit the range of policy instruments available to governments. Procedural constriction: Occurs when the process of policy-making is limited or influenced. This may include regulatory chill, when the potential threat of trade sanctions or costly litigation deters national governments from initiating policy processes. It may also include transparency/notification or regulatory coherence mechanisms that bring new international actors and institutions into the domestic policy-making process. Structural constriction: Occurs if trade and investment policy facilitates a shift from public to private provision of goods and services such that the economic and regulatory power of private sector actors is expanded.

^[2]^ Notably, the first two of these agreements no longer exist: the NAFTA was renegotiated to the United States Mexico Canada Agreement (USMCA), and the TPPA never ratified but replaced with the Comprehensive and Progressive Agreement on Trans-Pacific Partnership (CPTPP) without United States’ membership. The literature gathered did not include any in-depth analyses of either of these new agreements.

